# SARS-CoV-2 in Nursing Homes: Analysis of Routine Surveillance Data in Four European Countries

**DOI:** 10.14336/AD.2022.0820

**Published:** 2023-04-01

**Authors:** Tristan Delory, Julien Arino, Paul-Emile Haÿ, Vincent Klotz, Pierre-Yves Boëlle

**Affiliations:** ^1^ Sorbonne Université, INSERM, Institut Pierre Louis d’Épidémiologie et de Santé Publique, IPLESP, F-75012, Paris, France.; ^2^Centre Hospitalier Annecy Genevois, France.; ^3^Department of Mathematics, University of Manitoba, Winnipeg, Manitoba, Canada.; ^4^Groupe Colisee.

**Keywords:** SARS-CoV-2, nursing homes, cohort, Europe, vaccination

## Abstract

Transmission of SARS-CoV-2 in nursing homes is poorly documented. Using surveillance data of 228 European private nursing homes, we estimated weekly SARS-CoV-2 incidences among 21,467 residents and 14,371 staff members, compared to that in the general population, between August 3, 2020, and February 20, 2021. We studied the outcomes of “episodes of introduction” where one case was first detected and computed attack rates, reproduction ratio (*R*), and dispersion parameter (*k*). Out of 502 episodes of SARS-CoV-2 introduction, 77.1% (95%CI, 73.2%-80.6%) led to additional cases. Attack rates were highly variable, ranging from 0.4% to 86.5%. The *R* was 1.16 (95%CI, 1.11-1.22) with *k* at 2.5 (95%CI, 0.5-4.5). The timing of viral circulation in nursing homes did not mirror that in the general population (*p*-values<0.001). We estimated the impact of vaccination in preventing SARS-CoV-2 transmission. Before vaccination’s roll-out, a cumulated 5,579 SARS-CoV-2 infections were documented among residents and 2,321 among staff. Higher staffing ratio and previous natural immunization reduced the probability of an outbreak following introduction. Despite strong preventive measures, transmission likely occurred, regardless of building characteristics. Vaccination started on January 15, 2021, and coverage reached 65.0% among residents, and 42.0% among staff by February 20, 2021. Vaccination yielded a 92% reduction (95%CI, 71%-98%) of outbreak probability, and lowered *R* to 0.87 (95%CI, 0.69-1.10). In the post-pandemic era, much attention will have to be paid to multi-lateral collaboration, policy making, and prevention plans.

Old age was found to be the main risk-factor for the severity of SARS-CoV-2 infection, prompting nursing homes to adopt strategies to prevent introduction and spread of the disease among residents [1-7]. Yet, it seems that mortality by COVID-19 was higher in elderly living in nursing homes. Such differences may be explained by repeated uncontrolled outbreaks in closed environments, but could also be a consequence of the poorer health status of nursing home residents compared to elderly living in the community [3, 4]. The huge diversity in nursing home organization and adoption of control measures adds to the difficulty in interpretation [8].

Most data about nursing homes was collected in early 2020, during the first wave of the pandemic, before national policies for prevention of viral introduction and spread went into effect [3, 5, 9-13]. Recently, vaccination reduced the likelihood of outbreaks and improved the outcome of infected residents [14-17].

Here, we use routinely collected surveillance data in private nursing homes during the first year of the pandemic to understand how SARS-CoV-2 introduction in nursing homes compared with circulation in the general population, and how the outcomes of introduction varied in periods with mild and strong interventions, including the vaccination.

## METHODS

Data originated from routine surveillance, collected daily in a centralized electronic database for 228 nursing homes in France (97), Belgium (73), Spain (45), and Italy (13) between March 13^th^, 2020, and February 20^th^, 2021. Data collection complied with the European General Data Protection Regulation (GDPR).

We compared the incidence of cases in time with that in the general population in each country. We defined an “episode of introduction” in a nursing home as a time period during which COVID-19 cases were detected in residents or staff with less than 14 days intervals between successive cases [18]. We computed the attack rate during an episode and determined whether the initial case of an episode was a resident and/or a staff member. We computed the percentage of successful introductions (episodes leading to at least one case). Finally, we estimated reproduction ratios (*R*) and dispersion parameter (*k*) using the distribution of attack rates [19, 20].

We analysed the probability of successful introduction in the pre- and post-vaccination era, determining factors associated with a higher probability of successful introduction, and the effect of increasing coverage in vaccination using multivariable logistic regressions.

All analyses were performed with R version 4.0.1. All tests were two-tailed, alpha 5% bilateral. Details for methods can be found in Supplementary materials.

**Figure 1.**
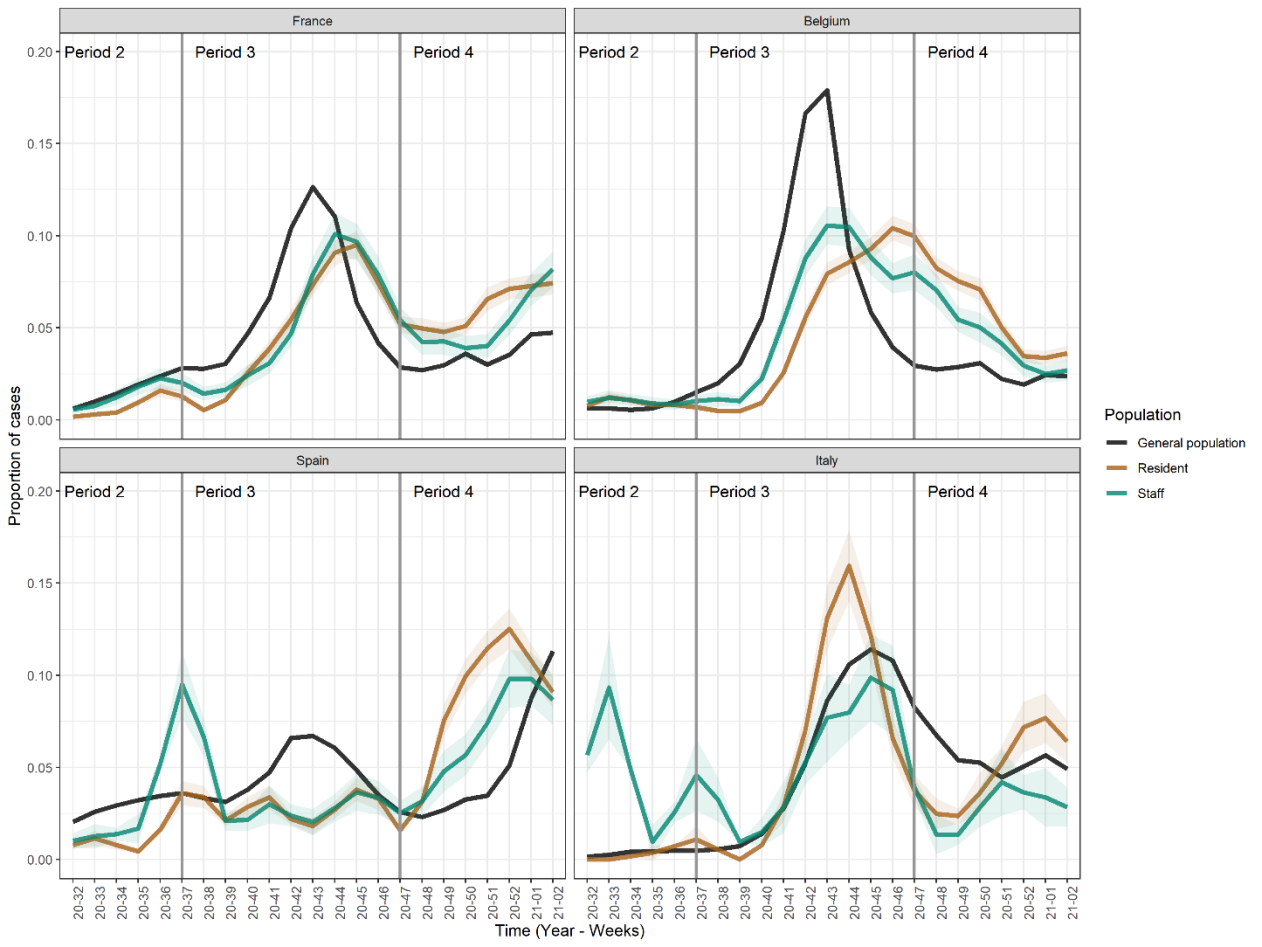
Time distribution of incident cases in nursing homes and in the general population by country. Residents (brown line), staff members (green line) are plotted using a 3-weeks weighted moving average for proportions. Shaded areas correspond to 95% confidence intervals. Black line represents the weekly proportion within the general population. In all countries except Spain, most cases were found in Period 3 for nursing home individuals and the general population.

## RESULTS

At the beginning of the pandemic, 21,467 residents were living and 14,371 staff members working in the surveyed facilities. Details in Supplementary materials.

Before vaccination’s roll-out, 5,579 SARS-CoV-2 infections were documented among residents (Supplementary Table 2). There were 3,966 residents newly admitted over the study period and 710 deaths from all causes, including COVID-19. The case-fatality ratio in SARS-CoV-2 infected residents was 12.7% (95%CI, 11.9%-13.6%). Infection was found in 2,321 staff members.

We found a positive correlation between daily incidences among residents and staff members (*r*=0.64 (95%CI, 0.53-0.74)). Although the general profile of incidence over time was similar, the overall timing of cases in facilities was not the same as in the general population (*p*-values<0.001, Fig. 1). Introducing lag distributions between the general population and nursing homes did not lead to a better agreement.

We identified 502 episodes of introduction, of which, at onset, 403 (80.3%) involved only residents, and 99 (19.7%) involved both residents and staff members; 405 (80.7%) occurred before vaccination started, and 97 (19.3%) afterwards. Overall, extra case was generated in 77.1% of episodes (95%CI, 73.2%-80.6%). The probability of successful introduction decreased after vaccination’s roll-out, from 76.8% (95%CI, 72.4%-80.6%) to 56.7% (95%CI, 46.8%-66.6%, *p*-value<0.001). Before vaccination, when introduction led to extra cases, median attack rate was 3.7% (IQR, 1.6%-18.4%), ranging from 0.4% to 86.5%. Afterwards, attack rate remained similar at 3.0% (IQR, 1.3%-13.1%).

After September 15^th^, 2021, 266 episodes of introduction started with a single case (212 before vaccination and 54 after), from which we estimated that *R* was 1.16 (95%CI, 1.11-1.22) before vaccination and dropped to 0.87 (95%CI, 0.69-1.10) afterwards (*p-value*<0.001) (Supplementary Fig. 3). The data supported heterogeneity in transmission as the 5% who transmitted the most accounted for 20% of all new infections before vaccination (*k*=3.3, 95%CI, 1.5-27.0); while it was 35% afterwards (*k*=0.56, 95%CI, 0.21-2.7). When immunization (previous infections or vaccination) was high, the attack rates following introduction were small (Supplementary Fig. 4).

An increase by 1% of the cumulative infection rate decreased the probability of successful introduction by 3% (95%CI, 2%-5%), and increase in staffing ratio of 10%, by 5 staff in average (SD±2), decreased the probability of successful introduction by 21% (95%CI, 7%-34%) (Supplementary Table 2).

The vaccination started between January 15^th^ and 21^st^ depending on the country. By the end of February, the overall median immunization rate, including previous infections or recent vaccination, was 82.9% (IQR, 64.7%-100.0%) for residents, and 55.0% (IQR, 36.1%-87.1%) for staff members (Supplementary Table 3). Among residents 2,217 infections occurred during vaccination roll-out and the case-fatality ratio in infected residents was 15.1% (95%CI, 13.7%-16.7%) (Supplementary Table 4). The probability of successful viral introduction decreased with increasing vaccine coverage, by 92% (95%CI, 71%-98%) one month after the roll-out started (Table 1).

**Table 1:** Effect of vaccination scaling-up on the probability of successful viral introduction.

Period	Failed N = 136	Successful N = 366	aOR*	95%CI	P-value
Before vaccination	94 (69.1%)	311 (85.0%)	Ref		
January 15 to January 31	12 (8.8%)	37 (10.1%)	0.89	0.42 - 1.92	0.770
February 01 to February 15	17 (12.5%)	14 (3.8%)	0.23	0.10 - 0.52	<0.001
February 16 to February 28	13 (9.6%)	4 (1.1%)	0.08	0.02 - 0.29	<0.001

* Adjusted on study period, country, staffing ratio, cumulative attack rate at onset of introduction, and number of PCR per 1000-residents or 1000-staff members, at onset of introduction, and nursing home maximal capacity.

## DISCUSSION

In European nursing homes that applied the same set of prevention measures, multiple episodes of introduction of SARS-CoV-2 occurred with heterogeneous outcomes. The *R* of SARS-CoV-2, in the presence of interventions to prevent transmission, was around 1.16 before vaccination and dropped to 0.87 afterwards. Higher staffing ratios and immunization (through infection or vaccination) reduced the probability of spread after viral introductions.

The measures implemented were typical: reduction of visits, increased hygiene to comply with regulatory requirements and even went beyond national guidelines with the weekly screening of staff members from November 2020 onward. Centralized surveillance assured the prospective collection of data, while its content changed over the modifications in policies and the availability of tests. At the beginning of the pandemic, tests were restricted to the confirmation of symptomatic cases, and it is probable that some infections were missed, as a larger portion of symptomatic cases occurred in Periods 1-2 (58%). Afterwards, testing increased, but the positivity rate among residents remained low, suggesting that extensive contact screening and tracing was carried out. If cases were missed, it would lead to overestimate the number of susceptible individuals and overlooked episodes of introduction, and in turn, lowering attack rates and *R* during episodes of transmission. To limit this effect, we focused on episodes of introductions detected once the systematic testing of staff members was going on and adopted a gap of 14 days to declare the end of an episode of introduction.

We did not find the same timing for cases in the general population and in nursing homes. This contrasts with observations from the first wave, and suggests that prevention measures limited the inflow of SAR-CoV-2 infected individuals from the general population [3, 12]. While no general pattern was obvious between the countries, cases in nursing homes appeared less concentrated around the peak of the second wave of the pandemic in Europe in Fall 2020. This is in line with limited introduction, but suggests that transmission occurred and remained possible in these closed environments irrespective of the general epidemic (*R* was slightly above 1) [21]. *R* was however much lower than initially reported, providing evidence that contact tracing and screening among staff members [22] and design of facilities helped limit the spread of infection [21]. The heterogeneity in transmission indicates that super-spreading was possible, even though less than generally reported for COVID-19 [23].

We found that higher staffing ratios and natural immunization reduced the likelihood of success for viral introduction before vaccination roll-out [11]. This advocates for an increase in staffing ratios as a reasonable way to prevent and reduce viral spread [11]. Only vaccination reduced *R* and the probability of successful viral introductions, up to 90% [15-17]. In the post-pandemic era, these observations will be of importance for elaborating future preventive measures, and design of facilities [13, 16, 24]. More granular data are needed to decipher the role of staff as potential sources of introductions.

Our study also has limitations. First, we use aggregated data, without information on age class or underlying conditions, necessitating to make conservative assumptions about initial susceptibility of individuals, thereby lowering the estimated value of all indicators, in particular the attack rate and *R*. Second, the definition of cases evolved over time, according to tests availability and testing strategies, which may have resulted in underestimating indicators, especially among asymptomatic cases [25]. Third, we did not analyse the first waves of the pandemic because of bulk reporting before August 2020 conversely to others [11, 21]. The period of analysis spanned the European second autumnal wave in the general population, when Health professionals and authorities were aware of effective measures for preventing infection, spread, and the clinical management of cases [8]. Therefore, we may have underestimated the effect of building characteristics on epidemic dynamic in the early days of the pandemic [26]. Also, the B.1.1.7. strain was not yet circulating on Europe mainland, and we could not investigate the role of SARS-CoV-2 variants [27-29]. Nevertheless, our data emphasizes that centralized decision and policy making, appropriate staffing ratio, and strong preventive measures are likely to be more important than configuration of buildings for preventing outbreaks [24, 30-32]. Finally, the follow-up was too short to estimate the waning immunity [33].

In conclusion, SARS-CoV-2 introductions in European nursing homes led to mixture of epidemics with limited super-spreading events, despite the strong preventive measures designed after the first wave of the pandemic. No specific pattern of building characteristic mitigated this risk. High staffing ratio, natural immunization and vaccination were very efficient in preventing further epidemics. In the post-pandemic era, much attention will have to be paid to multi-lateral collaboration, policy making, and prevention plans.

## Supplementary Materials

The Supplementary data can be found online at: www.aginganddisease.org/EN/10.14336/AD.2022.0820.
