# Supplementary material for: SARS-CoV-2 in Nursing Homes: Analysis of Routine Surveillance Data in Four European Countries
Source: Aging Dis. 2023 Apr 1;14(2):325–30. doi: 10.14336/AD.2022.0820 (PMC10017157; doi:10.14336/AD.2022.0820)
Supplement: Supplementary file 1 — The Supplementary data can be found online at: www.aginganddisease.org/EN/10.14336/AD.2022.0820. [file AD-14-2-325-s.pdf]

SUPPLEMENTARY DATA

SARS-CoV-2 in Nursing Homes: Analysis of Routine Surveillance Data in Four European Countries

Tristan Delory[1,2*], Julien Arino[3], Paul-Emile Haÿ[4], Vincent Klotz[4], Pierre-Yves Boëlle[1]

© 2022. Delory T et al. Published online at http://www.aginganddisease.org/EN/10.14336/AD.20220820

SUPPLEMENTARY DATA

METHODS

Data collected and control measures

Data originated from routine surveillance, collected daily in a centralized electronic database for 228 nursing homes in France, Belgium, Spain, and Italy between March 13th, 2020, and February 20th, 2021. In this period, the overall mean daily number of residents was 22,406 (SD ± 1,029) and the mean number of staff was 12,596 (SD ± 1,608). Each participating nursing home contributed data to a centralized electronic database at the head office of the Colisee group, the fourth largest commercial care home operator in Europe. Data quality was assessed daily by remote monitoring and inconsistencies were resolved prospectively. Data collection complied with the European General Data Protection Regulation (GDPR). Only anonymized daily counts were available for analysis.

The data included the number of COVID-19 cases, the number of tests for residents and staff, and their results. For residents, number of hospitalisations, deaths of all-cause, death among SARS-CoV-2 infected, and number of new admissions, whereas for staff, it was the number of sick leaves and exclusion. COVID-19 surveillance changed over time due to regulatory decisions, and the availability of tests. Over the first sixth months of the pandemic, until August 3rd, 2020, only symptomatic cases in patients and staff were tested for COVID-19. Starting in August 2020, contact tracing and testing around test-positive residents was carried out; and this was extended to staff one month later (September 2020). Weekly screening of staff members using PCR tests was initiated in November 2020, at the same time when the ban on visits was lifted. Vaccination started on January 15th, 2021 using two doses of mRNA BNT162b2 vaccine provided at 21 days intervals.

For each participating nursing home, we collected the number of beds and rooms and computed a staff to resident ratio.

Supplementary Table 1. Testing policy and strategies implemented in nursing homes for preventing COVID-19 transmission over the study period.

<table>
<tr><th>Measures</th><th>Period 1 (P1)
Before
W33 - 2020</th><th>Period 2 (P2)
W33-20
to W37-20</th><th>Period 3 (P3)
W37–20
to W47–20</th><th>Period 4 (P4)
W47–20
to W02–21</th><th>Period 5 (P5)
Since
W02–21</th></tr>
<tr><td>Contact tracing</td><td></td><td></td><td></td><td></td><td></td></tr>
<tr><td>Residents</td><td></td><td style="background:gray"></td><td style="background:gray"></td><td style="background:gray"></td><td style="background:gray"></td></tr>
<tr><td>Staff members</td><td></td><td></td><td style="background:gray"></td><td style="background:gray"></td><td style="background:gray"></td></tr>
<tr><td>Weekly PCR screening</td><td></td><td></td><td></td><td></td><td></td></tr>
<tr><td>Residents</td><td></td><td></td><td></td><td></td><td></td></tr>
<tr><td>Staff members</td><td></td><td></td><td></td><td style="background:gray"></td><td style="background:gray"></td></tr>
<tr><td>Vaccination</td><td></td><td></td><td></td><td></td><td></td></tr>
<tr><td>Residents</td><td></td><td></td><td></td><td></td><td style="background:gray"></td></tr>
<tr><td>Staff members</td><td></td><td></td><td></td><td></td><td style="background:gray"></td></tr>
<tr><td>No visits</td><td style="background:gray"></td><td style="background:gray"></td><td style="background:gray"></td><td></td><td></td></tr>
<tr><td>Negative PCR before visits</td><td></td><td></td><td></td><td style="background:gray"></td><td style="background:gray"></td></tr>
</table>

Temporal counts with respect to COVID-19 infection

We computed the weekly incidence of COVID-19 cases in each nursing home for residents and staff. We compared the observed distribution of cases in time with that in the general population in each country using *Chi-squared tests*. We described the status of participants and reconstructed the temporal counts with respect to COVID-19 as either susceptible to infection or removed from the chain of transmission after being infected or vaccinated from August 2020 to January 2021. We computed the number of participants susceptible to COVID-19 at the beginning of August 2020 by subtracting the cumulated number of reported COVID-19 infections over the period March-July 2020. Then, we updated the number of susceptible individuals daily by removing participants from the count upon infection or 36 days after receiving a first vaccine dose. For residents, we also removed those who died during the study period or

© 2022. Delory T et al. Published online at http://www.aginganddisease.org/EN/10.14336/AD.20220820

went to hospital. As the status of newly admitted residents was unknown, they were added to the count as being susceptible to infection.

Definition of an episode of introduction

We defined an "episode of introduction" in a nursing home as a time period during which COVID-19 cases were detected in residents or staff with less than 14 days intervals between successive cases. The 14 days duration corresponds to the maximum gap between individuals in the same chain of transmission, and has been recommended as the quarantine duration for COVID-19.[19] Some episodes saw both resident and staff cases detected on the same day. By construction, each episode of introduction started with a case detected at least 14 days after the previous case in the same nursing home and ended with the first gap of at least 14 days between successive detections (Supplementary **Error! Reference source not found.**). There could be several episodes of transmission in the same nursing home, but s uccessive episodes did not overlap.

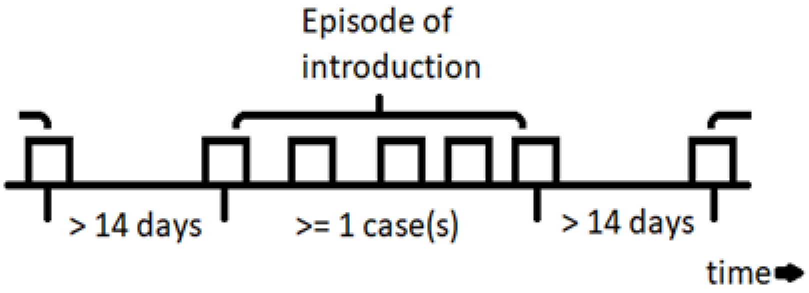

Supplementary Figure 1. Definition of an episode of introduction in a nursing home. Within the episode, successive cases are detected less than 14 days apart.

Attack rate (*AR*), and reproduction ratio (*R*)

We obtained from our previous computations the initial number of cases (*i0*) and of susceptible individuals (*s0*) at the beginning of each episode of introduction, and computed the number of cases during each episode (*Z*). We computed the attack rate during an episode as ($AR = Z/s0$). We determined whether the initial case of an episode was a resident and/or a staff member; some episodes saw both resident and staff cases detected on the same day. We computed the percentage of successful introductions as the percentage of episodes of introduction leading to at least one case ($Z \geq 1$).

We estimated reproduction ratios (*R*) in successive periods using the distribution of attack rates computed in episodes of introduction.[20] We allowed for negative binomial dispersion in the number of secondary cases as suggested by earlier reports.[21] We only analysed episodes of introduction starting after September 15th, when contact tracing was generalized (Period 3, Supplementary Table S1). We tested for a change in *R* value at two time points: when routine PCR testing in staff started (Period 4), then when vaccination started (Period 5).

Probability of successful introduction - Multivariable logistic regression

We analysed the probability of successful introduction in the pre- and post-vaccination era. We first determined factors associated with a higher probability of successful episodes of introduction in the pre-vaccination period using a multivariable logistic regression including country, active screening for staff members (yes/no), staffing ratio ($N_{staff\ working}/N_{residents\ in\ NH}$), cumulative attack rate (0%-100%) and bed capacity of nursing homes at onset of introduction. We also adjusted for the number of tests among residents and staff (tests per 1000-residents/staff) at the beginning of the episode.

We then analysed jointly episodes pre- and post-vaccination, using the same variables as above and including time: before vaccination, then 3 half-month periods starting January 15th and ending February 28th corresponding to increasing coverage in vaccination.

© 2022. Delory T et al. Published online at http://www.aginganddisease.org/EN/10.14336/AD.20220820

SUPPLEMENTARY DATA

RESULTS

Characteristics of nursing homes

The median bed capacity of nursing homes was 88 (IQR, 74-120), with 16 (IQR, 0-28) dementia beds and 68% of facilities with at least one multiple occupancy bedroom. In France, nursing homes were smaller with the least multiple occupancy bedrooms. In Italy, all facilities were divided into « residents' nuclei », units comprising shared apartments. The staffing ratio was similar across countries at 0.7 (IQR, 0.6-0.8).

Tests for SARS-CoV-2 among residents and staff members

The percentage of residents tested for SARS-CoV-2 infection was above 10% with changes over time and by country. The positivity rate of tests performed for SARS-CoV-2 among residents was between 0% and 17.6%. Among staff members, the percentage tested was below 5% before the systematic weekly screening and contact tracing (Period 1-3), and increased above 50% afterwards (Period 4, Supplementary Figure S2). After implementation of systematic weekly screening among staff members, the positivity rate decreased sharply below 10%.

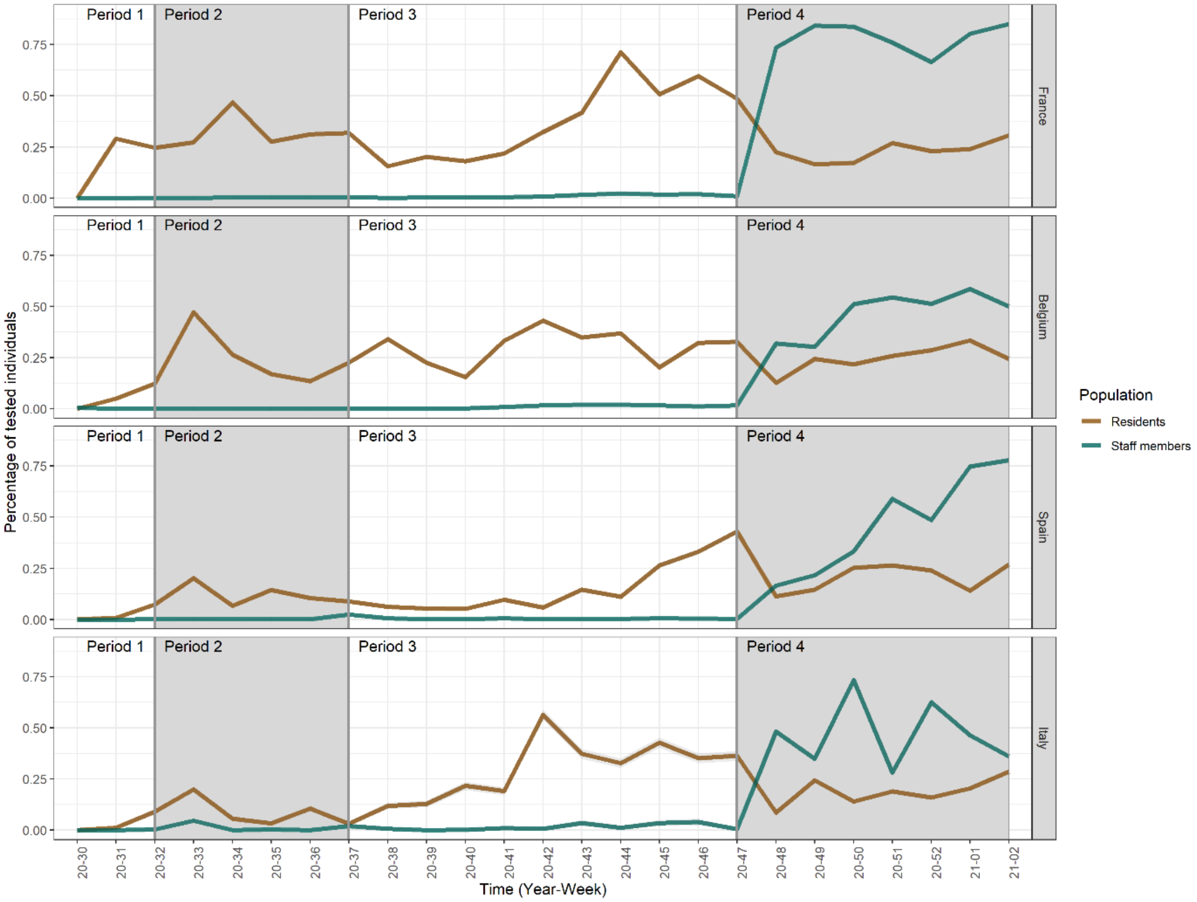

Supplementary Figure 2. Weekly percentage of individuals tested in nursing home, by country, in the period before vaccination. Change is shown according to population type. Vertical regions correspond to major changes in testing policy and strategy for controlling epidemics, as described in Table 1.

© 2022. Delory T et al. Published online at http://www.aginganddisease.org/EN/10.14336/AD.20220820

SUPPLEMENTARY DATA

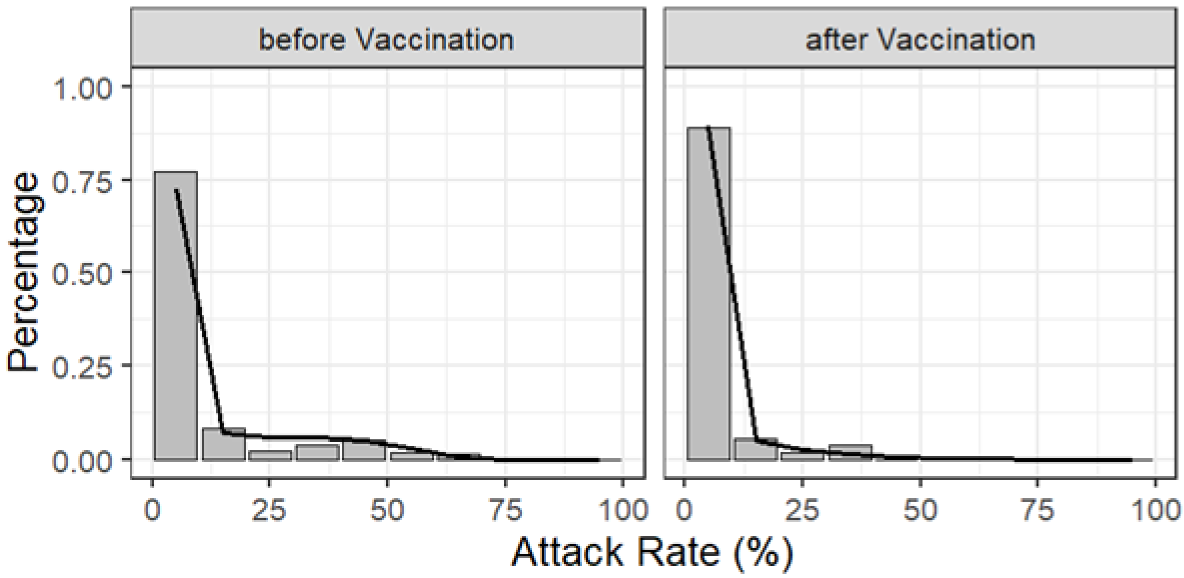

Supplementary Figure 3: Distribution of attack rates during episodes of introduction in nursing homes, before and after the roll-out of vaccination. Bars are observed attack rates (n=212 episodes of introduction before rollout and n=56 after rollout). Lines are model predicted attack rates distribution using best fit parameters.

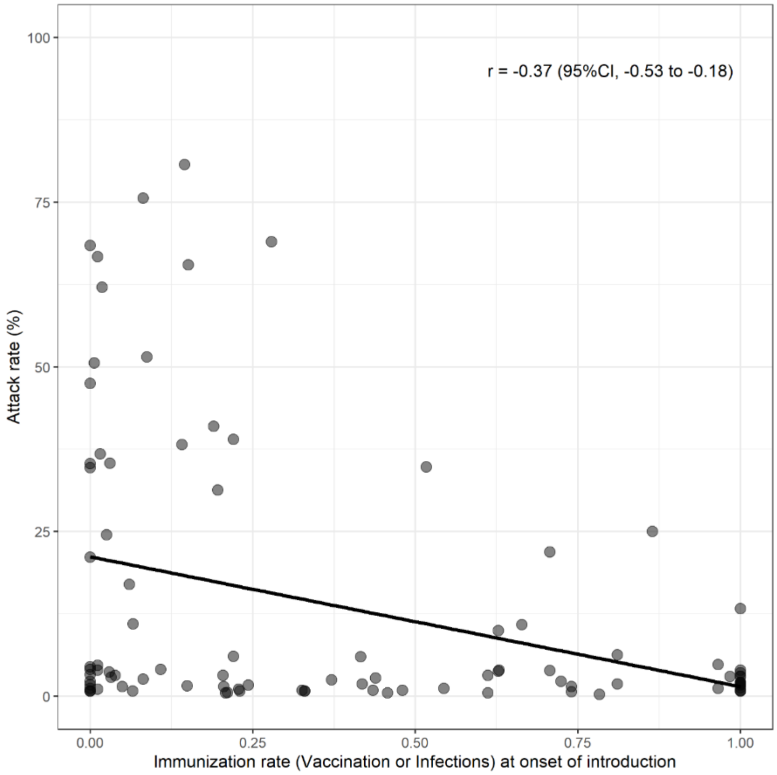

Supplementary Figure 4. Attack rate as a function of immunization (previous infection or vaccination) after the roll-out of vaccination. Black line represents the linear regression line, correlation coefficient r = -0·37 (95%CI, -0·53 to -0·18).

© 2022. Delory T et al. Published online at http://www.aginganddisease.org/EN/10.14336/AD.20220820

SUPPLEMENTARY DATA

Supplementary Table 2. Factors associated with successful episodes of SARS-CoV-2 introduction among Nursing Home individuals (residents or staff members) before the scaling-up of vaccination.

Factors	Unsuccessful introduction N = 96	Successful introduction N = 316	aOR*	95%CI	*P*-value
Staffing ratio (per 0.1 increase)	0.56 (SD±0.19)	0.52 (SD±0.16)	0.79	0.66 – 0.93	0.005
Nursing Home maximal capacity (per 20-bed increase)	107 (SD±37.4)	109 (SD±40.1)	1.09	0.93 – 1.27	0.304

* Adjusted odds ratio. The value is obtained by multivariable analysis adjusted on study period, country, staffing ratio, cumulative attack rate at onset of introduction, and number of PCR per 1000-residents or 1000-staff members, at onset of introduction, and nursing home maximal capacity.

Supplementary Table 3. Immunization by vaccination and previous infection, among nursing home residents and staff members, by country and overall, 45 days after vaccination started.

Country	Residents		Staff members	
	Vaccination (V) *median [IQR]*	Vaccination or Previous Infection *median [IQR]*	Vaccination (V) *median [IQR]*	Vaccination or Previous Infection *median [IQR]*
France	52.4% [27.7%, 68.1%]	68.6% [57.3%, 86.3%]	15.9% [8.5%, 29.8%]	41.5% [26.9%, 53.7%]
Belgium	73.1% [64.7%, 83.1%]	98.9% [76.9%, 100%]	72.3% [43.8%, 81.5%]	73.4% [46.7%, 100%]
Spain	69.5% [59.6%, 81.9%]	93.3% [79.6%, 100%]	78.8% [69.9%, 87.0%]	86.3% [60.0%, 98.6%]
Italy	38.5% [16.6%, 69.7%]	75.7% [38.5%, 100%]	17.9% [4.4%, 71.0%]	55.1% [31.1%, 84.3%]
Overall	65.0% [42.9%, 76.0%]	82.9% [64.7%, 100%]	42.0% [14.4%, 76.5%]	55.0% [36.1%, 87.1%]

Supplementary Table 4. Number of SARS-CoV-2 cases among residents, including symptomatic infections, those requiring oxygen support, transfers to hospitals, and deaths, before and after the roll-out of vaccination.

Indicator	Before vaccination †		After vaccination (Period 5)
	Without contact tracing around staff (Periods 1 and 2)	*With contact tracing around staff (Periods 3 and 4)*	
Cases among residents*	N = 3,083	N = 2,496	N = 2,152
Symptomatic cases	58.1% (n = 1,791)	38.1% (n = 950)	41.6% (n = 896)
Oxygen support	21.9% (n = 675)	19.8% (n = 494)	18.7% (n = 402)
Transfer to hospital	14.9% (n = 459)	14.3% (n = 357)	14.9% (n = 320)
Death among cases	11.2% (n = 344)	14.7% (n = 366)	14.7% (n = 316)

* Number of residents diagnosed with SARS-CoV-2 infection.
† Testing policy evolved over time, before the scaling-up of vaccination. Notably, contact tracing around infected staff members started on week 48.
Periods are referring to measures implemented by head office across the nursing home. Periods and measures are detailed in Supplementary Table 1.

© 2022. Delory T et al. Published online at http://www.aginganddisease.org/EN/10.14336/AD.20220820
